# 5-Fluorouracil Induced Intestinal Mucositis via Nuclear Factor-κB Activation by Transcriptomic Analysis and *In Vivo* Bioluminescence Imaging

**DOI:** 10.1371/journal.pone.0031808

**Published:** 2012-03-07

**Authors:** Chung-Ta Chang, Tin-Yun Ho, Ho Lin, Ji-An Liang, Hui-Chi Huang, Chia-Cheng Li, Hsin-Yi Lo, Shih-Lu Wu, Yi-Fang Huang, Chien-Yun Hsiang

**Affiliations:** 1 Emergency Medicine Department, Far Eastern Memorial Hospital, Taipei, Taiwan; 2 Department of Life Sciences, National Chung Hsing University, Taichung, Taiwan; 3 Graduate Institute of Chinese Medicine, China Medical University, Taichung, Taiwan; 4 Department of Nuclear Medicine, China Medical University, Taichung, Taiwan; 5 Department of Radiation Therapy and Oncology, China Medical University Hospital, Taichung, Taiwan; 6 School of Pharmaceutical Sciences and Chinese Medicine Resources, China Medical University, Taichung, Taiwan; 7 Department of Biochemistry, China Medical University, Taichung, Taiwan; 8 Department of Prosthodontics, Chang Gung Memorial Hospital, Taoyuan, Taiwan; 9 Department of Microbiology, China Medical University, Taichung, Taiwan; Dr. Margarete Fischer-Bosch Institute of Clinical Pharmacology, Germany

## Abstract

5-Fluorouracil (5-FU) is a commonly used drug for the treatment of malignant cancers. However, approximately 80% of patients undergoing 5-FU treatment suffer from gastrointestinal mucositis. The aim of this report was to identify the drug target for the 5-FU-induced intestinal mucositis. 5-FU-induced intestinal mucositis was established by intraperitoneally administering mice with 100 mg/kg 5-FU. Network analysis of gene expression profile and bioluminescent imaging were applied to identify the critical molecule associated with 5-FU-induced mucositis. Our data showed that 5-FU induced inflammation in the small intestine, characterized by the increased intestinal wall thickness and crypt length, the decreased villus height, and the increased myeloperoxidase activity in tissues and proinflammatory cytokine production in sera. Network analysis of 5-FU-affected genes by transcriptomic tool showed that the expression of genes was regulated by nuclear factor-κB (NF-κB), and NF-κB was the central molecule in the 5-FU-regulated biological network. NF-κB activity was activated by 5-FU in the intestine, which was judged by *in vivo* bioluminescence imaging and immunohistochemical staining. However, 5-aminosalicylic acid (5-ASA) inhibited 5-FU-induced NF-κB activation and proinflammatory cytokine production. Moreover, 5-FU-induced histological changes were improved by 5-ASA. In conclusion, our findings suggested that NF-κB was the critical molecule associated with the pathogenesis of 5-FU-induced mucositis, and inhibition of NF-κB activity ameliorated the mucosal damage caused by 5-FU.

## Introduction

5-Fluorouracil (5-FU) is the most commonly used chemotherapy drug in the clinical oncologic practice. It is widely used for the treatment of various cancers, including gastrointestinal cancer, breast cancer, and head and neck cancer [1], [2]. Clinical evidence from patients undergoing 5-FU therapy indicates that personal response to 5-FU is different. Some people display slight side effects, while others suffer from severe adverse effects that lead to the discontinuance of cancer therapy. The commonly side effects of 5-FU include myelosuppression, dermatitis, cardiac toxicity, diarrhea, and mucositis [1], [3]. Among these adverse effects, gastrointestinal mucositis is a major complication that occurs in approximately 80% of patients receiving 5-FU and results in abdominal bloating as well as vomiting and diarrhea [4].

Mucositis usually appears along the entire gastrointestinal tract from mouth to anus and causes general debility. Mucositis of the intestine is characterized by increased crypt apoptosis and villus atrophy, leaving the mucosal tissue open to ulceration and infection [5]–[7]. Several factors or genes contributing to the 5-FU-induced mucositis have been studied. For examples, increased apoptosis and decreased cellularity by 5-FU cause the histological change in the intestine [8]. The formation of reactive oxygen species (ROS) and the production of proinflammatory cytokines, such as interleukin-1β (IL-1β), IL-6 and tumor necrosis factor-α (TNF-α), lead to the mucosal damage [9]–[11]. Additionally, the production of platelet-activating factor (PAF) participates in the pathogenesis of mucositis [12]. Although several genes have been suggested to be involved in the 5-FU-induced intestinal mucositis, the key molecules, especially the upstream transcription factors that regulate the downstream genes associated with the pathogenesis of mucositis are still uncertain. Moreover, better compounds targeting to the mechanism of mucosal injury remain to be developed for the treatment of mucositis.

Nuclear factor-κB (NF-κB) is an inducible transcription factor that consists of heterodimers of RelA (p65), c-Rel, RelB, p50/NF-κB1, and p52/NF-κB2. NF-κB is a central coordinator of innate and adaptive immune responses. It is also involved in the regulation of inflammatory cytokine production and inflammation [13], [14]. When cells are exposed to the inflammatory and stress stimulators, NF-κB activates the downstream genes, including cytokine, cytokine receptor and cyclooxygenase genes, resulting in the inflammatory process [15], [16]. Accordingly, controlling NF-κB activation may be a potent strategy for the treatment of inflammation.

In this study, we analyzed the mechanism of 5-FU-induced intestinal damage by transcriptomic analysis and bioluminescent imaging. Our findings demonstrated that NF-κB was the likely key molecule involved in the 5-FU-induced mucositis and inhibition of NF-κB activity by 5-aminosalicylic acid (5-ASA) improved the mucosal damage caused by 5-FU.

## Materials and Methods

### Animal experiments

Mouse experiments were conducted under ethics approval from China Medical University Animal Ethics Committee (Permit Number: 97-28-N). Transgenic mice carrying the NF-κB-driven luciferase gene were constructed as described previously [17]. BALB/c mice were purchased from National Laboratory Animal Center (Taipei, Taiwan).

The 5-FU-induced mucositis model was established as described previously [7]. Male mice (6 to 8 weeks old) were injected intraperitoneally with phosphate-buffered saline (PBS) (137 mM NaCl, 1.4 mM KH_2_PO_4_, 4.3 mM Na_2_HPO_4_, 2.7 mM KCl, pH 7.2) or 5-FU (100 mg/kg) (Sigma, St Louis, MO, USA). For 5-ASA treatment, mice were orally administered with 5-ASA (130 mM/kg) for two consecutive days before intraperitoneal administration of 5-FU and two consecutive days after 5-Fu administration. Mice were imaged for the luciferase activity or sacrificed for histological and immunohistochemical evaluations at indicated periods.

### Histological analysis

Mice intestines were fixed in 10% phosphate-buffered formalin for 2 d and dehydrated in a series of graded alcohols (50%, 70%, and 95%) for 30 min each. Samples were then embedded in paraffin, cut into 5-µm sections, stained with hematoxylin and eosin (H&E), and subjected to blinded histological assessment. Villus height was measured from the baseline to the villus tip. Crypt length was measured from the baseline to the submucosa. The thickness of intestinal wall was measured from the submucosa to the serosa. Three independent measurements from 3 different longitudinal sections per mouse were made.

### Microarray analysis

Total RNA was extracted from jejunum using RNeasy Mini kit (Qiagen, Valencia, CA, USA). Total RNA was quantified and evaluated as described previously [18]. Microarray analysis was performed as described previously [18]–[20]. Briefly, fluorescent-labeled RNA targets were prepared from 5 µg of total RNA using MessageAmp™ aRNA kit (Ambion, Austin, TX, USA) and Cy5 dye (Amersham Pharmacia, Piscataway, NJ, USA). Fluorescent targets were hybridized to the Mouse Whole Genome OneArray™ (Phalanx Biotech Group, Hsinchu, Taiwan) and scanned by an Axon 4000 scanner (Molecular Devices, Sunnyvale, CA, USA). Three replicates from three independent mice were performed. The Cy5 fluorescent intensity of each spot was analyzed by genepix 4.1 software (Molecular Devices). The signal intensity of each spot was corrected by subtracting background signals in the surroundings. We filtered out spots that signal-to-noise ratio was less than 0 or control probes. Spots that passed these criteria were normalized by R program [21]. The fold changes of genes were calculated by dividing the normalized signal intensities of genes in 5-FU-treated mice by those in PBS-treated mice. Genes with fold changes ≥2 or ≤−2 were selected and used as the input genes for the generation of biological network using Transcription Regulation algorithm in MetaCore™ Analytical suite (GeneGo Inc., St. Joseph, MI, USA). All microarray data are MIAME compliant and the raw data have been deposited in a MIAME compliant database (Gene Expression Omnibus, accession number GSE28873).

### *In vivo* and *ex vivo* imaging of luciferase activity

For *in vivo* imaging, mice were anesthetized with isoflurane and injected intraperitoneally with 150 mg/kg D-luciferin. Five minutes later, mice were placed facing up in the chamber and imaged for 1 min with the camera set at the highest sensitivity by IVIS Imaging System® 200 Series (Xenogen, Hopkinton, MA, USA). Photons emitted from the whole bodies were quantified using Living Image® software (Xenogen). Signal intensity was quantified as the sum of all detected photon counts from the whole body and presented as photon/sec. For *ex vivo* imaging, mice were anesthetized and injected with luciferin intraperitoneally. Five minutes later, mice were sacrificed and tissues were rapidly removed. Tissues were placed in the IVIS system and imaged with the same setting used for *in vivo* studies. Signal intensity was quantified as the sum of all detected photon counts per second within the region of interest after subtracting the background luminescence and presented as photon/sec/cm^2^/steradian (photon/sec/cm^2^/sr).

### Immunohistochemical staining

Sections of 5 µm were deparaffinized in xylene and rehydrated in graded alcohol. Endogenous peroxidase was quenched with 3% hydrogen peroxide in methanol for 15 min and the nonspecific binding was blocked with 1% bovine serum albumin at room temperature for 1 h. Sections were incubated with mouse monoclonal antibody against NF-κB p65 subunit (Chemicon, Temecula, CA, USA) or rabbit polyclonal antibody against IL-1β (Santa Cruz, CA, USA) or TNF-α (Abcam®, Cambridge, UK) at 1∶50 dilution overnight at 4°C and then incubated with biotinylated secondary antibody (Zymed Laboratories, Carlsbad, CA, USA) at room temperature for 20 min. Finally, slides were incubated with avidin-biotin complex reagent and stained with 3,3′-diaminobenzidine according to manufacturer's protocol (Histostains®-Plus, Zymed Laboratories).

### Cytokine enzyme-linked immunosorbent assay (ELISA)

IL-1β and TNF-α in sera were quantified by ELISA with Quantikine® mouse immunoassay kits (R&D Systems, Minneapolis, MN, USA). Briefly, sera were added to wells, which were coated with monoclonal antibody against IL-1β or TNF-α. After five washes, the biotinylated antibody against IL-1β or TNF-α, the peroxidase-conjugated avidin, and the chromogenic substrates were sequentially added to each well. The absorbance was read at 450 nm in an ELISA reader.

### Myeloperoxidase (MPO) assay

MPO activities in the jejunum were quantified with MPO colorimetric activity assay kit (BioVision, Mountain View, CA, USA). Briefly, the frozen tissues were homogenized and centrifuged to remove insoluble materials. Supernatants were collected, mixed with MPO assay buffer and MPO substrate, incubated at room temperature for 1 h, and then mixed with tetramethylbenzidine probe. The absorbance was read at 412 nm in an ELISA reader.

### Statistical analysis

Data were presented as mean ± standard error. Student's *t*-test was used for comparisons between two experiments. A value of *p*<0.05 was considered statistically significant.

## Results

### 5-FU induced intestinal mucositis

**Figure 1:**
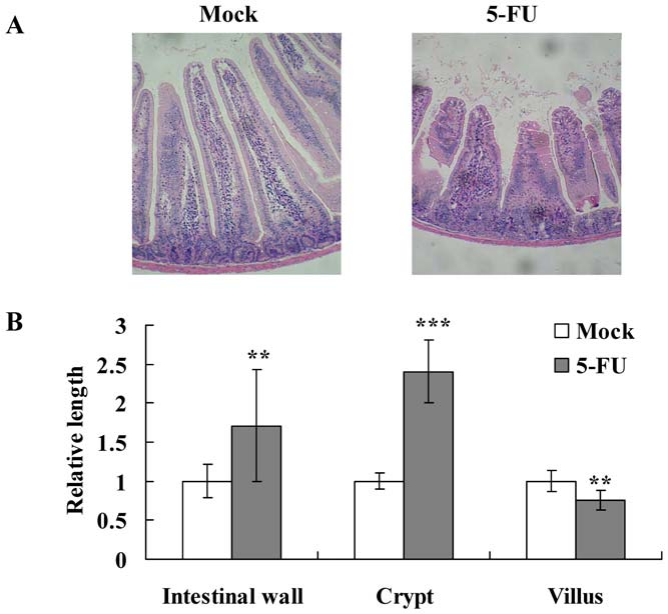
Histological examination of the small intestine following 5-FU administration. BALB/c mice were intraperitoneally administered with PBS (mock) or 5-FU and sacrificed 2 days later. (A) Microscopic features of the jejunum. Sections were stained with H&E and observed using light microscopy. Magnification 100×. Photos are representative images. (B) Intestinal morphometry of intestinal wall thickness, crypt length, and villus height. Six mice in each group were sacrificed for the morphometry analysis. Three intestinal walls, crypts, and villi in 3 longitudinal sections per mouse were counted. Results are expressed as relative length, which is presented as a comparison with the length or thickness relative to mock. Values are mean ± standard error. **p<0.01, ***p<0.001, compared with mock.

Chemotherapy-induced diarrhea occurs in approximately 80% of patients treated with 5-FU. Previous studies have shown that 5-FU kills progenitor cells in the crypts of Lieberkühn and the bases of villi, leading to the breakdown of mucosal barrier [22]. Moreover, 5-FU administration results in increased apoptosis and decreased cellularity in the small intestine [8]. We therefore intraperitoneally administered mice with 5-FU and the histological changes in the small intestine were evaluated 2 days later. In comparison with mock, 5-FU caused mucosal damage in the small intestine (Figure 1). 5-FU decreased the height of villi and caused the blunting and fusion of villi. Moreover, 5-FU led to the intestinal inflammation, characterized by the infiltration of immune cells and the accumulation of fluid, and subsequently increased the length of crypts. 5-FU also increased the thickness of intestinal wall. These findings indicated that intraperitoneal administration of 5-FU caused the mucosal damage and inflammation in the small intestine.

### NF-κB was the central molecule in the 5-FU-affected gene expression network

**Figure 2:**
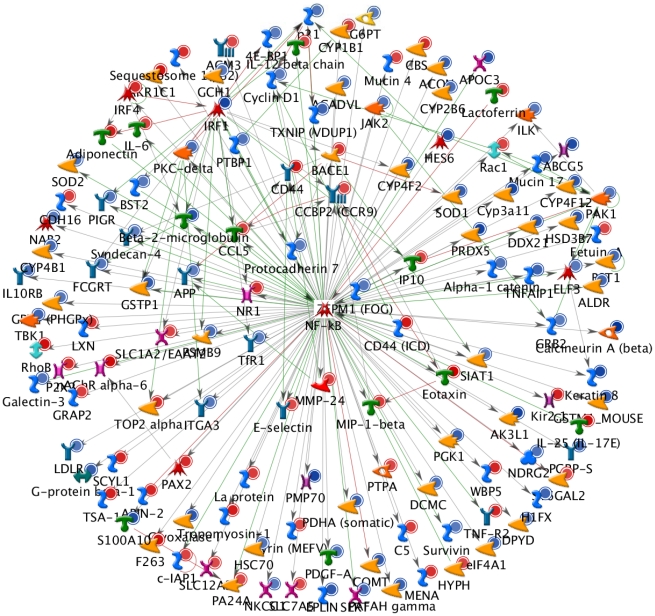
Network analysis of 5-FU-affected genes in the small intestine. Upregulated genes are marked with red circles/disks. Downregulated genes are marked with blue circles/disks. Cyan lines indicate the fragments of canonical pathways.

We further elucidated the mechanism of 5-FU-induced intestinal mucositis by transcriptomic analysis. In a total of 29,922 genes, 1,614 genes were upregulated and 1,574 genes were downregulated by 2 fold by 5-FU. These genes were selected for the generation of biological network using Transcription Regulation algorithm in MetaCore. As shown in Figure 2, 5-FU-affected genes were directly connected to the NF-κB, suggesting that expressions of 5-FU-affected genes were regulated by NF-κB. The expression levels of genes in the network are shown in Table S1. Furthermore, NF-κB seemed to be the central molecule of the network. These findings suggested that NF-κB was the likely key molecule involved in the 5-FU-induced intestinal mucositis.

### 5-FU evoked the NF-κB activity judged by *in vivo* and *ex vivo* imaging

Transcriptomic analysis showed that NF-κB was the central molecule in the 5-FU-affected gene expression network. We therefore performed *in vivo* and *ex vivo* imaging to elucidate the NF-κB activity in mice following 5-FU administration. Transgenic mice carrying the luciferase gene driven by a promoter with five NF-κB responsive elements were used here. The luciferase activity reflected the NF-κB *trans*-activity.

**Figure 3:**
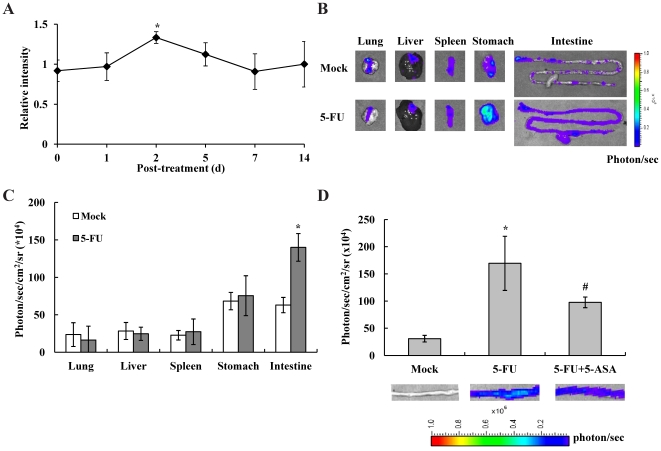
NF-κB-dependent bioluminescence in living mice and individual organs following 5-FU administration. (A) *In vivo* imaging. Transgenic mice were injected intraperitoneally with PBS or 5-FU and imaged at indicated periods. Results are expressed as relative intensity, which is presented as the comparison with the NF-κB-dependent bioluminescent signal relative to mock. Values are mean ± standard error (*n* = 6 per group). *p<0.05, compared with mock. (B) *Ex vivo* imaging. Transgenic mice were injected intraperitoneally with PBS (mock) or 5-FU. Two days later, mice were sacrificed and organs were subjected to image. The color overlay on the image represents the photon/sec emitted from the organs, as indicated by the color scale. Photos are representative images (*n* = 6 per group). (C) Quantification of photon emission from the organs. Values are mean ± standard error (*n* = 6 per group). **p<0.01, compared with mock. (D) NF-κB-dependent bioluminescence in the intestine following 5-FU and/or 5-ASA administration. Transgenic mice were administered with 5-FU and/or 5-ASA and imaged 2 days later. The color overlay on the image represents the photon/sec emitted from the intestine, as indicated by the color scale. Photos are representative images (*n* = 6 per group). Quantification of photon emission from the intestine was shown on the top. Values are mean ± standard error. *p<0.05, compared with mock. ^#^ *p*<0.05, compared with 5-FU treatment.

Transgenic mice were intraperitoneally given with PBS or 5-FU, and the bioluminescent imaging was performed on 0, 1, 2, 5, 7, and 14 d. Figure 3A shows that a maximal induction of NF-κB activity was observed on 2 d following 5-FU administration and *ex vivo* imaging was therefore performed on 2 d. As shown in Figure 3B and Figure 3C, 5-FU slightly affected the NF-κB activities in lung, liver, spleen, and stomach, while 5-FU significantly activated the NF-κB activity in the small intestine by 2.2-fold. These findings indicated that 5-FU evoked the whole body NF-κB activity on 2 d and induced the NF-κB activation in the small intestine. Moreover, 5-FU-induced intestinal mucositis could be assessed by NF-κB bioluminescent imaging.

### 5-FU-induced NF-κB activity was inhibited by 5-ASA

**Figure 4:**
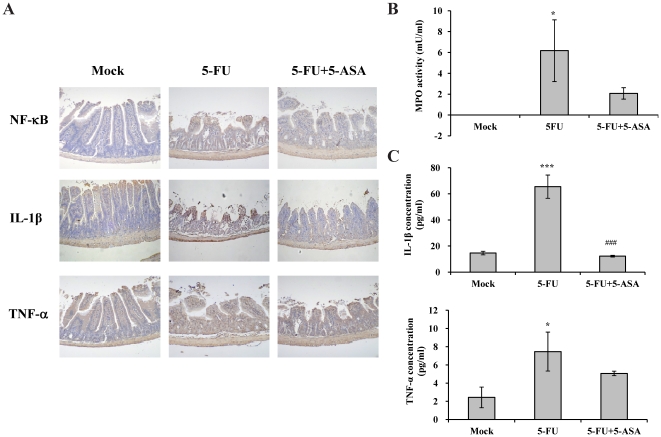
Immunohistochemical staining and MPO activity of jejunum and cytokine ELISA of sera following 5-FU and/or 5-ASA administration. Transgenic mice were administered with 5-FU and/or 5-ASA and sacrificed 2 days later. (A) Sections were stained by immunohistochemistry using antibody against NF-κB, IL-1β, or TNF-α. Magnification 100×. Photos are representative images (*n* = 6 per group). (B) MPO activity assay. Frozen jejunum was homogenized and MPO activity in the tissue was analyzed. Values are mean ± standard error. *p<0.05, compared with mock. (C) Cytokine ELISA. The levels of IL-1β and TNF-α were analyzed by cytokine ELISA. Values are mean ± standard error. *p<0.05, ***p<0.001, compared with mock. ^###^ *p*<0.001, compared with 5-FU treatment.

5-ASA is an anti-inflammatory drug that has been used for the treatment of ulcerative colitis for decades [23]. Activation of NF-κB in biopsies of ulcerative colitis is suppressed by 5-ASA, suggesting that 5-ASA is a potent inhibitor of NF-κB activation *in vivo* [23]. We therefore evaluated whether 5-ASA inhibited 5-FU-induced NF-κB activation and subsequently ameliorated the 5-FU-caused mucositis. Transgenic mice were administered with 5-FU and/or 5-ASA and imaged 2 days later. 5-FU induced the NF-κB activity in the small intestine, which was in agreement with aforementioned findings (Figure 3D). However, 5-ASA significantly reduced the 5-FU-induced NF-κB activity, with a 42% reduction of bioluminescent intensity. Immunohistochemical staining with antibody against NF-κB p65 subunit revealed that, in comparison with mock, there were many brown p65-reactive cells in the crypts and villi of 5-FU-treated intestine (Figure 4A). However, 5-ASA reduced the number of brown p65-reactive cells in the intestine. These findings indicated that 5-FU evoked the NF-κB activity, while 5-ASA inhibited 5-FU-induced NF-κB activity in the intestine.

### Inhibition of NF-κB activity ameliorated the 5-FU-induced mucositis in the small intestine

**Figure 5:**
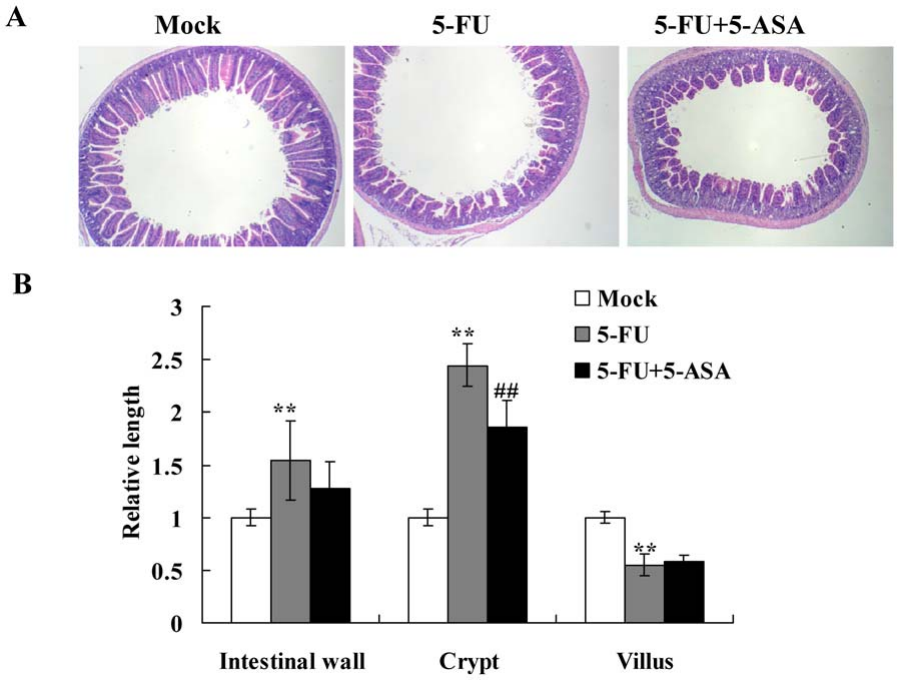
Histological examination of the small intestine following 5-FU and/or 5-ASA administration. Transgenic mice were administered with 5-FU and/or 5-ASA and sacrificed 2 days later. (A) Microscopic features of the jejunum. Sections were stained with H&E and observed using light microscopy. Magnification 40×. Photos are representative images (*n* = 6 per group). (B) Intestinal morphometry of intestinal wall thickness, crypt length, and villus height. Six mice in each group were sacrificed for the morphometry analysis. Three intestinal walls, crypts, and villi in 3 longitudinal sections per mouse were counted. Results are expressed as relative length, which is presented as a comparison with the length or thickness relative to mock. Values are mean ± standard error. **p<0.01, compared with mock. ^##^ *p*<0.01, compared to 5-FU treatment.

We further tested whether the inhibition of 5-FU-induced NF-κB activity by 5-ASA improved the 5-FU-caused intestinal mucositis. Histological examination of the small intestine following 5-FU and/or 5-ASA treatment showed that 5-FU increased the thickness of intestinal wall and the length of crypt, while 5-ASA significantly decreased 5-FU-caused histological changes (Figure 5). 5-FU also decreased the height of villus, while 5-ASA slightly increased it. In addition to the histological changes, MPO activities in the intestine were induced by 5-FU and suppressed by 5-ASA, also indicating that 5-FU induced intestinal inflammation, while 5-ASA suppressed 5-FU-induced inflammation (Figure 4B). The levels of IL-1β and TNF-α in the tissues and sera were evaluated by immunohistochemical staining and cytokine ELISA, respectively. As shown in Figures 4A and 4C, 5-FU induced the immunomarcation for IL-1β and TNF-α in the tissues and increased the levels of IL-1β and TNF-α in sera, while 5-ASA suppressed 5-FU-induced IL-1β and TNF-α production in the tissues and sera. These findings suggested that 5-FU induced intestinal mucositis via NF-κB activity. Moreover, inhibition of NF-κB activity decreased the 5-FU-induced TNF-α production and subsequently improved the 5-FU-caused mucosal damage in the small intestine.

## Discussion

5-FU is a commonly used chemotherapy drug for the treatment of malignant tumors. It kills tumor cells through interfering DNA synthesis and affecting protein synthesis [2]. Approximately 80% of patients undergoing 5-FU therapy suffer from a range of symptoms, including mucositis and diarrhea. Gastrointestinal mucositis is frequently associated with pain and increased risk of infection. It leads to impaired quality of life in patients. Moreover, patients may no longer be able to continue cancer therapy in cases of severe mucositis [24]. Therefore, developing better therapeutic drug targeting to the mechanisms of mucosal damage is awaited.

Mechanisms involved in the pathogenesis of mucositis are very complex. Apoptosis, hypoproliferation, and inflammation contribute to the mucosal injury [9]. It has been reported that the expression of proinflammatory cytokines, such as IL-6 and TNF-α, in the small intestine and colon of rodents after chemotherapy is significantly increased [7], [25]. IL-1 and IL-1 receptor antagonist are produced locally in the intestinal mucosa, and their expressions are increased in inflammatory mucosa [26], [27]. Moreover, IL-1β plays a critical role in the genesis and development of intestinal mucositis after chemotherapy, and this type of effect is caused by inducing crypt cell apoptosis [11]. In addition to the proinflammatory cytokines, ROS generated by inducible nitric oxide synthase (iNOS) and cyclooxygenase-2 (COX-2) lead to the mucosal injury. Increased iNOS and COX-2 activity in the 5-FU- and radiation-induced mucositis, suggesting the important role of ROS in the pathogenesis of oral mucositis [9], [10]. Recently, the role of PAF in 5-FU-induced intestinal mucositis has been suggested using knockout animals and an antagonist of PAF receptor [12]. Because the expressions of proinflammatory cytokines, iNOS, COX-2, and PAF are regulated by various transcription factors, we applied transcriptomic analysis to find the upstream transcription factors that regulate the downstream gene expression and lead to mucosal injury.

Transcriptomic analysis by DNA microarray tool is a popular research and screening tool for differentially expressed genes. Microarray-based gene expression patterns have been used to predict the clinical outcome and prognosis of patients undergoing 5-FU therapy [28]-[30]. It has also been applied to predict the therapeutic efficacy of 5-FU and to identify the biomarkers in various cancers [31], [32]. We used microarray tool for the first time to identify the key molecule involved in the 5-FU-caused intestinal injury in this study. The expression levels of IL-6, TNF-α, and IL-1β were increased, with fold changes of 2.28, 3.37, and 6.77, respectively (data not shown). These data were in agreement with previous reports. Further network analysis using Transcription Regulation algorithm indicated that the expression of 5-FU-affected genes was regulated by NF-κB, and NF-κB was the central molecule in the biological network. These findings suggested that NF-κB was the upstream key molecule that regulated the expression of downstream genes and led to the mucositis of intestine.

NF-κB is a central coordinator of innate and adaptive immune responses. NF-κB has also been linked to the control of cell growth, apoptosis, and cell cycle [33]. Previous reports have implicated the NF-κB in the pathogenesis of several inflammatory diseases, such as local joint inflammation, glomerulonephritis, and inflammatory bowel diseases [34]-[36]. NF-κB activation is also found in biopsy tissues in cancer patients treated with radiation and several chemotherapeutic drugs, except 5-FU [6], [37]. As a consequence of the gene upregulation by the initial activation of NF-κB, a broad range of biological active proteins accumulate and target to the submucosa tissue in the gastrointestinal tract. NF-κB activation induced by anti-neoplastic agents and radiation is therefore though to elicit the inflammatory and apoptotic responses that lead to the mucosal injury. In this study, we found that NF-κB was the critical molecule that regulated the expression of 5-FU-affected genes, and NF-κB activity was induced by 5-FU in the intestine. In contrast, other studies indicated that 5-FU administration inhibits NF-κB activation *in vitro*. Aota et al [38] and Azuma et al [39] reported that 5-FU suppresses NF-κB activity via the inhibition of IκB kinase activity and subsequently induces apoptosis in human salivary gland cancer cells. Contradictory effects of NF-κB activation on normal and cancer cells have been reported [40]. Activation of NF-κB can be either pro-apoptotic or anti-apoptotic, depending on the target cells. Therefore, it is possible that NF-κB activated by 5-FU results in apoptotic signals and proinflammatory cytokine production in normal mucosal tissue and sequentially contributed to the injury of gastrointestinal tract.

Bioluminescent imaging was applied to evaluate the NF-κB activity after 5-FU administration. Transgenic mice carrying the luciferase gene under the control of NF-κB-responsive element were constructed previously, and the bioluminescent signal correlated with NF-κB activity indicated that bioluminescent intensity represents NF-κB activity *in vivo* [17], [36]. Oral administration of 5-ASA has been used for decades for the treatment of inflammatory bowel disease [23]. 5-ASA is an anti-inflammatory drug that inhibits NF-κB activation and suppressed the inflammatory response [23]. In this study, we also found that 5-ASA decreased 5-FU-induced NF-κB activity and immunomarcation for IL-1β and TNF-α in the intestine. The histological changes of mucositis have also been improved. These findings suggested that inhibition of NF-κB activity might result in the suppression of inflammation and the sequential amelioration of mucositis in the intestine.

In conclusion, our findings suggested that NF-κB was the critical molecule involved in the 5-FU-caused mucosal injury, while inhibition of NF-κB activity suppressed the 5-FU-induced inflammation and sequentially improved the 5-FU-induced mucosal damage. These findings suggested that NF-κB was the potent target for the development of drugs for the treatment of 5-FU-induced mucositis.

## Supporting Information

Table S1 **Expression levels of genes in the network in 5-FU-induced mucositis.**
(DOC)
