# Supplementary material for: 5-Fluorouracil Induced Intestinal Mucositis via Nuclear Factor-κB Activation by Transcriptomic Analysis and In Vivo Bioluminescence Imaging
Source: PLoS One. 2012 Mar 7;7(3):e31808. doi: 10.1371/journal.pone.0031808 (PMC3296709; doi:10.1371/journal.pone.0031808)
Supplement: Table S1 — Expression levels of genes in the network in 5-FU-induced mucositis. (DOC) [file pone.0031808.s001.doc]

Table S1 Expression levels of genes in the network in 5-FU-induced mucositis.

| Gene_symbol | Gene_description | Fold change^a^ | *p* value^b^ |
| --- | --- | --- | --- |
| Ccl11 | Small chemokine (C-C motif) ligand 11 (Eotaxin) | 5.39±0.79 | 4.2×10^-5^ |
| Chrna6 | Cholinergic receptor, nicotinic, alpha polypeptide 6 | 4.84±5.74 | 0.18105 |
| Il25 | Interleukin 25 | 4.66±5.72 | 0.24311 |
| Rhob | Ras homolog gene family, member B | 4.61±6.28 | 0.38070 |
| Pfkfb3 | 6-Phosphofructo-2-kinase/fructose-2,6-biphosphatase 3 | 4.30±2.90 | 0.11444 |
| Akr1c21 | Aldo-keto reductase family 1, member C21 | 4.23±2.93 | 0.02762 |
| Adipoq | Adipocyte-specific protein AdipoQ | 3.79±2.65 | 0.15414 |
| Tpm1 | Tropomyosin 1, alpha | 3.77±1.74 | 0.01850 |
| Ly6e | Lymphocyte antigen 6 complex, locus E | 3.59±0.45 | 5.4×10^-5^ |
| Kcnj2 | Potassium inwardly-rectifying channel, subfamily J, member 2 | 3.55±3.46 | 0.14500 |
| Tnip2 | TNFAIP3 interacting protein 2 | 3.44±3.35 | 0.15565 |
| Scyl1 | SCY1-like 1 (*S. cerevisiae*) | 3.41±1.32 | 0.01068 |
| Wbp5 | WW domain binding protein 5 | 3.39±1.33 | 0.00927 |
| Tnfrsf1b | Tumor necrosis factor receptor superfamily, member 1b | 3.37±3.94 | 0.32369 |
| Pax2 | Paired box gene 2 | 3.12±3.34 | 0.26449 |
| Irf4 | Interferon regulatory factor 4 | 3.10±2.07 | 0.15196 |
| P2rx2 | Purinergic receptor P2X, ligand-gated ion channel, 2 | 3.07±1.17 | 0.01485 |
| Sqstm1 | Sequestosome 1 | 3.04±3.33 | 0.31461 |
| Top2a | Topoisomerase (DNA) II alpha | 3.03±1.96 | 0.04543 |
| Cbs | Cystathionine beta-synthase | 3.03±2.83 | 0.20677 |
| Enah | Enabled homolog (*Drosophila*) | 2.95±2.75 | 0.49872 |
| Grin1 | Glutamate receptor, ionotropic, NMDA1 (zeta 1) (NR1) | 2.85±2.19 | 0.11010 |
| Lxn | Latexin | 2.79±3.07 | 0.36349 |
| Bace1 | Beta-site APP cleaving enzyme 1 | 2.71±1.37 | 0.08244 |
| Ltf | Lactotransferrin | 2.64±3.11 | 0.48107 |
| Ccl4 | Chemokine (C-C motif) ligand 4 (MIP-1-beta) | 2.52±2.40 | 0.29653 |
| Hc | Hemolytic complement (C5) | 2.51±1.32 | 0.10525 |
| Ssb | Sjogren syndrome antigen B | 2.49±2.39 | 0.29861 |
| Cdh16 | Cadherin 16 | 2.42±1.04 | 0.02407 |
| Cyp1b1 | Cytochrome P450, family 1, subfamily b, polypeptide 1 | 2.37±1.02 | 0.02609 |
| Grap2 | GRB2-related adaptor protein 2 | 2.35±1.06 | 0.03017 |
| Ccl5 | Chemokine (C-C motif) ligand 5 | 2.34±1.05 | 0.03075 |
| Cxcl10 | Chemokine (C-X-C motif) ligand 10 (ip10) | 2.34±1.05 | 0.03076 |
| Il6 | Interleukin 6 | 2.28±1.38 | 0.14487 |
| Rac1 | RAS-related C3 botulinum substrate 1 | 2.27±2.06 | 0.61901 |
| Mmp24 | Matrix metallopeptidase 24 | 2.23±0.57 | 0.00509 |
| Slc1a2 | Solute carrier family 1 (glial high affinity glutamate transporter), member 2 | 2.21±0.91 | 0.04296 |
| Muc4 | Mucin 4 | 2.21±1.23 | 0.08205 |
| Ppp2r4 | Protein phosphatase 2A, regulatory subunit B (PR 53) | 2.18±1.59 | 0.23068 |
| Zdhhc17 | Zinc finger, DHHC domain containing 17 | 2.17±1.20 | 0.11078 |
| Ahsg | Alpha-2-HS-glycoprotein | 2.14±1.18 | 0.11679 |
| Ccbp2 | Chemokine binding protein 2 | 2.14±1.31 | 0.16119 |
| Chrm3 | Cholinergic receptor, muscarinic 3, cardiac | 2.13±0.86 | 0.03724 |
| Pla2g4a | Phospholipase A2, group IVA (cytosolic, calcium-dependent) | 2.10±1.37 | 0.18116 |
| Sele | Selectin, endothelial cell | 2.07±0.82 | 0.04074 |
| Slc12a3 | Solute carrier family 12, member 3 | 2.05±1.34 | 0.19739 |
| St6gal2 | Beta galactoside alpha 2,6 sialyltransferase 2 | 2.04±0.98 | 0.11112 |
| Slc7a5 | Solute carrier family 7 (cationic amino acid transporter, y+ system), member 5 | 2.04±0.81 | 0.04360 |
| Mefv | Mediterranean fever | 2.03±1.53 | 0.25031 |
| Il12b | Interleukin 12b | 2.02±1.54 | 0.26543 |
| App | Amyloid beta (A4) precursor protein | -2.00±0.59 | 0.01319 |
| Sdc4 | Syndecan 4 | -2.01±0.15 | 8.8×10^-5^ |
| Tfrc | Transferrin receptor (TfR1) | -2.03±0.82 | 0.02629 |
| Pcdh7 | Protocadherin 7 | -2.06±0.08 | 7.2×10^-6^ |
| Grb2 | Growth factor receptor bound protein 2 | -2.06±0.15 | 8.1×10^-5^ |
| G6pc | Glucose-6-phosphatase, catalytic | -2.07±0.10 | 1.7×10^-5^ |
| Slc12a2 | Solute carrier family 12, member 2 | -2.10±0.40 | 0.00223 |
| Eif4ebp1 | Eukaryotic translation initiation factor 4E binding protein 1 | -2.11±0.22 | 0.00024 |
| Dpyd | Dihydropyrimidine dehydrogenase | -2.13±0.31 | 0.00099 |
| Gch1 | GTP cyclohydrolase 1 | -2.16±0.16 | 6.8×10^-5^ |
| Birc5 | Baculoviral IAP repeat-containing 5 | -2.16±0.16 | 6.1×10^-5^ |
| St6gal1 | Beta galactoside alpha 2,6 sialyltransferase 1 (SIAT1) | -2.17±0.26 | 0.00045 |
| Bst2 | Bone marrow stromal cell antigen 2 | -2.23±0.12 | 1.3×10^-5^ |
| Ilk | Integrin linked kinase | -2.23±0.39 | 0.00122 |
| Zfpm1 | Zinc finger protein, multitype 1 | -2.24±0.49 | 0.00329 |
| Itga3 | Integrin alpha 3 | -2.35±0.10 | 4.2×10^-6^ |
| Fcgrt | Fc receptor, IgG, alpha chain transporter | -2.36±0.15 | 2.2×10^-5^ |
| Gpx4 | Glutathione peroxidase 4 | -2.36±1.85 | 0.06871 |
| Ldlr | Low density lipoprotein receptor | -2.37±0.33 | 0.00046 |
| Pak1 | P21 (CDKN1A)-activated kinase 1 | -2.40±0.34 | 0.00038 |
| Cdkn1a | Cyclin-dependent kinase inhibitor 1A (P21) | -2.45±0.04 | 4.5×10^-8^ |
| Comt | Catechol-O-methyltransferase | -2.45±0.26 | 0.000146 |
| Pigr | Polymeric immunoglobulin receptor | -2.49±2.08 | 0.06463 |
| Sod2 | Superoxide dismutase, mitochondrial precursor | -2.53±0.44 | 0.00091 |
| Ctnna1 | Catenin alpha-1 | -2.54±0.57 | 0.00209 |
| Acadvl | Acyl-Coenzyme A dehydrogenase, very long chain | -2.56±0.34 | 0.00024 |
| Ptbp1 | Polypyrimidine tract binding protein 1 | -2.56±0.14 | 7.3×10^-6^ |
| Lgals3 | Lectin, galactose binding, soluble 3 | -2.57±1.20 | 0.03328 |
| Ndrg2 | N-myc downstream regulated gene 2 | -2.64±0.14 | 3.9×10^-6^ |
| Mlycd | Malonyl-CoA decarboxylase | -2.70±0.11 | 2.3×10^-6^ |
| Psmb9 | Proteosome subunit, beta type 9 | -2.81±0.36 | 0.00016 |
| Il10rb | Interleukin 10 receptor, beta | -3.06±0.25 | 1.9×10^-5^ |
| Prkcd | Protein kinase C, delta | -3.09±0.23 | 1.3×10^-5^ |
| Slc6a4 | Solute carrier family 6 (neurotransmitter transporter, serotonin), member 4 | -3.11±0.24 | 1.6×10^-5^ |
| Bst1 | Bone marrow stromal cell antigen 1 | -3.14±0.54 | 0.00043 |
| Cyp4b1 | Cytochrome P450, family 4, subfamily b, polypeptide 1 | -3.21±0.12 | 8.4×10^-7^ |
| Pglyrp1 | Peptidoglycan recognition protein 1 | -3.43±0.33 | 2.4×10^-5^ |
| H1fx | H1 histone family, member X | -3.57±0.46 | 6.9×10^-5^ |
| Txnip | Thioredoxin interacting protein | -3.60±0.65 | 0.00024 |
| Tbk1 | TANK-binding kinase 1 | -3.60±0.44 | 5.7×10^-5^ |
| Tnfaip1 | Tumor necrosis factor, alpha-induced protein 1 (endothelial) | -3.66±0.39 | 3.2×10^-5^ |
| Eif4a1 | Eukaryotic translation initiation factor 4A1 | -3.77±0.33 | 1.3×10^-5^ |
| Muc3 | Mucin 3, intestinal | -3.83±0.45 | 4.5×10^-5^ |
| Pdha1 | Pyruvate dehydrogenase E1 alpha 1 | -3.87±0.43 | 3.1×10^-5^ |
| Pafah1b3 | Platelet-activating factor acetylhydrolase, isoform 1b, alpha1 subunit | -3.98±0.38 | 1.5×10^-5^ |
| Aco2 | Aconitase 2, mitochondrial | -4.22±2.20 | 0.00547 |
| Sod1 | Superoxide dismutase 1, soluble | -4.23±2.70 | 0.01145 |
| Pgk1 | Phosphoglycerate kinase 1 | -4.36±1.49 | 0.00188 |
| Gnb1 | Guanine nucleotide binding protein, beta 1 | -4.42±1.75 | 0.00198 |
| Gstm1 | Glutathione S-transferase, mu 1 | -4.65±1.88 | 0.00239 |
| Cyp4f14 | Cytochrome P450, family 4, subfamily f, polypeptide 14 | -4.77±3.12 | 0.03197 |
| Lima1 | LIM domain and actin-binding protein 1 | -4.83±0.47 | 1.1×10^-5^ |
| Glo1 | Glyoxalase 1 | -5.24±0.61 | 1.7×10^-5^ |
| Cyp4f13 | Cytochrome P450, family 4, subfamily f, polypeptide 13 | -5.29±0.63 | 1.6×10^-5^ |
| Apoc3 | Apolipoprotein C-III | -5.77±2.10 | 0.00109 |
| B2m | Beta-2 microglobulin | -5.78±6.05 | 0.02204 |
| Hes6 | Hairy and enhancer of split 6 (*Drosophila*) | -6.11±0.52 | 3.4×10^-6^ |
| S100a10 | S100 calcium binding protein A10 (calpactin) | -6.53±1.28 | 9.9×10^-5^ |
| Abcg5 | ATP-binding cassette, sub-family G (WHITE), member 5 | -6.99±0.61 | 2.8×10^-6^ |
| Cyp3a11 | Cytochrome P450, family 3, subfamily a, polypeptide 11 | -7.35±0.48 | 7.8×10^-7^ |
| Krt8 | Keratin, type II cytoskeletal 8 | -7.95±2.47 | 0.00031 |
| Irf1 | Interferon regulatory factor 1 | -8.15±1.27 | 2.2×10^-5^ |
| Prdx5 | Peroxiredoxin 5 | -8.29±1.71 | 5.0×10^-5^ |
| Abcd3 | ATP-binding cassette, sub-family D (ALD), member 3 (PMP70) | -8.51±1.32 | 1.6×10^-5^ |
| Gstp1 | Glutathione S-transferase, pi 1 | -9.88±4.95 | 0.00150 |

^a^ Values are mean ± standard error (*n*=3).

^b^ *p* values were calculated by the geneSetTest function implemented in the limma package.
